# The Predictive Value of Inter Arm Blood Pressure Difference, Inter Leg Blood Pressure Difference and Ankle Brachial Index for Acute Aortic Dissection

**DOI:** 10.31083/j.rcm2401009

**Published:** 2023-01-04

**Authors:** Quan Zhou, Sufang Huang

**Affiliations:** ^1^Tongji Hospital, Tongji Medical College, Huazhong University of Science and Technology, 430030 Wuhan, Hubei, China

**Keywords:** acute aortic dissection, extremities blood pressure, ankle-brachial index, inter-arm systolic blood pressure difference, inter-leg systolic blood pressure difference, prediction, prognosis

## Abstract

**Introduction:**

Abnormal inter arm systolic blood pressure, inter leg systolic blood pressure and ankle brachial index (ABI) are related to vascular diseases. Our aim was to evaluate the correlation of inter arm systolic blood pressure difference (IASBPD), inter leg systolic blood pressure difference (ILSBPD), and ABI with acute aortic dissection (AAD) and their role in predicting AAD.

**Methods:**

In this prospective case-control study, 180 patients with AAD admitted to the emergency department were prospectively and consecutively collected in Tongji Hospital from October 2019 to December 2020. 180 healthy people matched by sex, age and BMI served as control group. All participants were adults over 18 years of age who underwent four-limb blood pressure measurements. IASBPD, ILSBPD and ABI were compared between the two groups and their associations with AAD were analyzed.

**Results:**

A total of 360 patients (180 cases and 180 controls) were analyzed. In case group IASBPD was larger [(15.23 ± 16.15) mm Hg *vs.* (4.19 ± 3.63) mm Hg] and ILSBPD was larger (13.00 mm Hg *vs.* 5.70 mm Hg). ABI was smaller [(0.98 ± 0.24) *vs.* (1.12 ± 0.09)], and the difference was statistically significant (all *p *< 0.05). According to the receiver operating characteristic curve (ROC), IASBPD ≥10 mm Hg (Sen 61.7%, Spe 88.9%), ILSBPD ≥13 mm Hg (Sen 50.6%, Spe 80.6%) and ABI ≤0.9 (Sen 53.3%, Spe 87.2%), showed significant correlation with AAD (all *p *< 0.001).

**Conclusions:**

Compared with healthy people, IASBPD and ILSBPD levels were higher and ABI levels were lower in patients with AAD. IASBPD ≥10 mm Hg and ILSBPD ≥13 mm Hg can be used as indicators for early screening of AAD, and IASBPD ≥10 mm Hg has better predictive value for the occurrence of AAD. In patients with typical chest pain, attention needs to be paid to measuring blood pressure in the extremities.

## 1. Introduction

Acute aortic dissection (AAD) is a serious cardiovascular emergency that threatens human life and health. As we all know, significant inter arm systolic blood pressure difference (IASBPD) is a typical sign of AAD [1]. IASBPD in AAD patients is usually due to the false lumen caused by aortic tear extending to the brachiocephalic artery or left subclavian artery, resulting in a decrease in blood flow in one side of the upper arm [2]. National Institute for Health and Care Excellence (NICE) and European Society of Cardiology (ESC) all consider the IASBPD of 15 mm Hg or larger as the threshold of additional cardiovascular risk [3, 4]. Recently, it was found that IASBPD ≥10 mm Hg was an indicator of unhealthy cardiovascular conditions, and the difference of 10 mm Hg was regarded as the normal upper limit of IASBPD [5]. Previous studies [1, 2, 3] focused on IASBPD did not collect and analyze AAD patients four-limb blood pressure differences. This study attempted to investigate the predictive value of IASBPD, inter leg systolic blood pressure difference (ILSBPD) and ankle brachial index (ABI) for AAD.

## 2. Methods

This study was a prospective case-control study, and a total of 180 patients with AAD who were first admitted to the emergency department of Tongji hospital from October 2019 to December 2020 were selected as the case group by convenience sampling method. A total of 180 healthy people with the same gender, age difference of ±5 years and BMI difference of ±5% in the physical examination center of the hospital and the case group were selected as the control group during the same period. Medical records of the control group were collected and peripheral vascular diseases were excluded. Inclusion criteria of the case group: all patients were diagnosed by computed tomographic angiography (CTA), magnetic resonance imaging (MRI) and other imaging examinations finding false lumen or free internal diaphragm, meeting the diagnostic criteria of Aortic Disease Diagnosis and Treatment Guidelines of European Society of Cardiology (2014 edition) [6]. Patients were admission within 14 days of onset; Patients were willing to participate and cooperate with the study and all inpatient medical records were completed; Patients with AAD who were hospitalized repeatedly or with traumatic aortic dissection or with peripheral vascular diseases were excluded.

### 2.1 Collecting Four-Limb Blood Pressure

Two OMRON electronic blood pressure monitors (HEM-FM31, J30, Japanese OMRON, Kyoto, Japan) were used for measurement. Recorded client’s name and hospital/medical numbers before measurements. At room temperature environment, all participants were told to breathe calmly, do not move and do not talk, and the blood pressure of both upper arms was measured simultaneously at first. Then the blood pressure of ankle artery of both lower limbs was measured at the same time. The mean value of 3 measurements was taken at an interval of 2 minutes. ABI, IASBPD and ILSBPD were calculated respectively. ABI = ankle artery systolic blood pressure/brachial artery systolic blood pressure. In this study, the lower ABI of the left and right sides was taken. IASBPD = absolute value of systolic pressure difference between left and right upper arm. ILSBPD = absolute value of systolic pressure difference between left and right ankle.

### 2.2 Data Analysis

Excel 2019 was used to establish the database, we double-checked the input data, and SPSS version 23.0 (IBM Corp., Armonk, NY, USA) was used for statistical analysis. Receiver operating characteristic curve (ROC) was drawn to evaluate the predictive value and prognostic value of ABI, IASBPD and ILSBPD for AAD, and the Area Under Curve (AUC) was compared. AUC >0.7 indicates that the diagnostic effect is good. The truncation value corresponding to the maximum approximation index is selected to obtain the corresponding sensitivity and specificity.

### 2.3 Quality Control

According to the research purpose, the inclusion and exclusion criteria were specified in the research design, and the research objects meeting the criteria were selected. To train operators related to limb blood pressure measurement, the operators are familiar with the measurement method, process and matters needing attention to ensure the reliability and accuracy of limb blood pressure measurement data; Electronic sphygmomanometers of the same model were used for all measurements to avoid deviation caused by different measuring instruments; All data will be checked and entered by two people, and a certain proportion of data will be checked randomly to ensure complete consistency and saved to Excel database for data analysis.

## 3. Results

Comparison of general data between AAD group and control group. Age of AAD group and control group [(53.19 ± 9.58) years *vs*. (51.30 ± 9.44) years], male (78.9% *vs*. 78.9%), BMI [${\mathrm{kg/m}}^{2}$ (24.80 ± 2.98) *vs*. (24.80 ± 2.90 ${\mathrm{kg/m}}^{2}$)], alcohol (63.3% *vs*. 62.2%), there were no significant differences (*p *> 0.05). Compared with the control group, the proportion of smoking (58.3% *vs*. 40.6%) was higher in AAD group, and the differences were statistically significant (*p <* 0.05). In the two groups, IASBPD [(15.23 ± 16.15) mm Hg *vs*. (4.19 ± 3.63) mm Hg], ILSBPD (13.00 mm Hg *vs*. 5.70 mm Hg) were significantly higher in AAD group than in control group. ABI [(0.98 ± 0.24) *vs*. (1.12 ± 0.09)] was significantly lower than that of control group, and the difference was statistically significant (*p *< 0.05) (Table 1).

**Table 1.** **Comparison of general data between the AAD group and the control group**.

Characteristics		AAD (n = 180)	Controls (n = 180)	t/$\chi^{2}$/Z value	*p* value
Age		53.19 ± 9.58	51.30 ± 9.44	1.884	0.060
Gender, n (%)				<0.001	1.000
	Male	142 (78.9)	142 (78.9)		
	Female	38 (21.1)	38 (21.1)		
BMI (${\mathrm{kg/m}}^{2}$)		24.80 ± 2.98	24.80 ± 2.90	0.025	0.980
Smoke, n (%)				11.379	0.001
	Yes	105 (58.3)	73 (40.6)		
	No	75 (41.7)	107 (59.4)		
Alcohol, n (%)				0.048	0.827
	Yes	114 (63.3)	112 (62.2)		
	No	66 (36.7)	68 (37.8)		
ABI		0.98 ± 0.24	1.12 ± 0.09	–7.183	<0.001
IASBPD (mm Hg)		15.23 ± 16.15	4.19 ± 3.63	8.948	<0.001
ILSBPD (mm Hg)		13.00 (5.00,28.75)	5.70 (2.40,10.70)	5.852	<0.001

ROC curves were drawn to analyze the predictive value of ABI, IASBPD and ILSBPD for AAD. As ABI was opposite to the predictive test of AAD, reciprocal processing was performed. The truncation value of ABI for predicting AAD was 0.90, AUC was 0.714, 95% confidence interval was 0.659~0.769, sensitivity was 53.3%, specificity was 87.2%. IASBPD predicted the truncation value of AAD was 10.00 mm Hg, AUC was 0.779, 95% confidence interval was 0.730~0.828, sensitivity was 61.7%, specificity was 88.9%. ILSBPD predicted AAD with truncation value of 13.00 mm Hg, AUC of 0.673, 95% confidence interval of 0.617~0.729, sensitivity of 50.6%, specificity of 80.6%. According to the results, the AUC of both ABI and IASBPD is greater than 0.7, indicating that ABI and IASBPD have high predictive value for AAD (Table 2).

**Table 2.** **Univariate association of ABI/IASBPD/ILSBPD and AAD**.

Variable	AUC	*SE*	95% CI	Sen%	Spe%	*p* value	Cut off
ABI	0.714	0.028	0.659∼0.769	53.3	87.2	<0.001	0.90
IASBPD	0.779	0.025	0.730∼0.828	61.7	88.9	<0.001	10.00
ILSBPD	0.673	0.029	0.617∼0.729	50.6	80.6	<0.001	13.00

## 4. Discussion

Some studies defined IASBPD significantly as IASBPD greater than 10~20 mm Hg [3, 4, 5, 7, 8], we found that IASBPD levels were higher in AAD patients compared with healthy people. This may be related to aorta lesions in AAD patients. Many patients with AAD develop significant IASBPD due to a tear of the aorta that extends into the false lumen caused by the brachiocephalic artery or the left subclavian artery, resulting in reduced blood flow on the upper arm side. Sung Wook Um *et al*. [9] included 111 patients with AAD and performed a 1:1 case-control study according to age and sex, suggesting that bilateral upper limb systolic pressure difference ≥20 mm Hg may be a useful predictor of AAD. We found that IASBPD ≥10 mm Hg could be a useful predictor of AAD and better than ILSBPD and ABI, the difference in IASBPD is less susceptible to atherosclerotic changes. In contrast to the findings with Sung Wook Um, it may because they included only patients who had a bilateral blood pressure measurement performed. Suspicion of AAD should be raised if patients with severe chest pain and bilateral systolic differential pressure ≥10 mm Hg is present.

Previous studies [1, 2, 3] only focused on the difference in blood pressure in upper limbs of AAD, or concluded the relationship between bilateral systolic blood pressure and AAD on the basis of observation without statistical analysis, which makes it difficult to know whether lower limb blood pressure is associated with AAD or whether it can better diagnose AAD by comparing lower limb blood pressure differences. We performed data analysis by collecting blood pressure of the lower extremities in patients with AAD and found that ILSBPD was not as effective in predicting AAD. Although tearing of the false lumen to the lower extremity arteries might cause the blood pressure significantly lower than that of the upper arm, possibly due to the fact that this type of patients is too severely ill to have limb blood pressure measurements or already deceased before admission, resulting in their not being included in the study. But patients with AAD still had significantly ILSBPD compared to healthy people.

Previous studies have found that ABI is closely related to atherosclerosis, which is the most common cause of vascular stenosis [10]. ABI is the simplest and most accurate noninvasive test for the diagnosis of peripheral artery disease [11]. Abnormally low systolic blood pressure indicates arterial stenosis or occlusion. In the emergency department, it is also of important diagnostic value for patients with unexplained chest and abdominal pain, lumbago, unexplained hypotension shock, and suspected AAD. Because AAD can affect the aorta and its branches in different parts of the aorta to varying degrees, there can be significant differences in blood pressure in the limbs. In this study, we found ABI is less valuable for predicting AAD than IASBPD. Arteriosclerosis obliterans (ASO) may also contribute to abnormal lower extremity blood pressure, abnormal ABI does not indicate possible AAD in this situation. The computational method of ABI is more complicated than IASBPD also limits its application.

Although ABI, IASBPD and ILSBPD appear to be somewhat associated with AAD, it does not appear to be a particular sign of AAD as many patients with peripheral vascular disease will present with abnormal ILSBPD and ABI. ILSBPD and ABI may be of limited use in diagnosing AAD. However, if AAD is diagnosed, it is still important to check for systolic blood pressure differences in the extremities. Dissection may lead to hypoperitoneum in the extremities, resulting in asymmetric blood pressure in the extremities blood pressure in patients with AAD should be the higher of the bilateral measurements, and attention should also be paid to differences in lower limb blood pressure in AAD, which may reflect lower limb hypoperfusion.

## 5. Limitations

The number of samples collected is relatively small, a few patients with particularly severe AAD need to brake strictly, we did not measure their lower limbs blood pressure. All samples come from the same hospital, lacking certain representativeness, which may affect the research results to a certain extent. We adopted the method of single sequential measurements of limbs, and perhaps blood pressure measured with simultaneous four-limb method is more precise than unilateral limb blood pressure measurement. When AAD patients are combined with ASO, this method is less useful. Furthermore, there can be patients with AAD that do not develop blood pressure differentials. In the future, multi-center studies with large samples should be carried out to verify the conclusions of this study.

## 6. Conclusions

Compared with healthy people, IASBPD and ILSBPD levels were higher and ABI levels were lower in patients with AAD. Four-limb blood pressure difference might have some clinical value for the early identification of AAD, we found IASBPD ≥10 mm Hg and ILSBPD ≥13 mm Hg can be used as indicators for early screening of AAD, and IASBPD ≥10 mm Hg has certain predictive value for the occurrence of AAD. Although Four-limb blood pressure measurement is very necessary for physical examination, few studies have measured four-limb blood pressure in people with suspected AAD [9, 11], we should pay attention to the four-limb blood pressure differences that occur in patients.

## Data Availability

The authors do not wish to share their data, because the project is still under research.

## Implications for Clinical Practice

1. Blood pressure measurement of extremity is fast, objective and easy to obtain. Extremity blood pressure should be measured in the ambulance before people arrive at the emergency room.

2. Extremity blood pressure measurement can help doctors identify people at high risk of vascular events and detect potentially life-threatening diseases early.

3. Extremity blood pressure measurement can help doctors discover the patient’s condition changes timely, which is conducive to the timely treatment of AAD.
